# Maternal mid-pregnancy C-reactive protein and risk of autism spectrum disorders: the early markers for autism study

**DOI:** 10.1038/tp.2016.46

**Published:** 2016-04-19

**Authors:** O Zerbo, M Traglia, C Yoshida, L S Heuer, P Ashwood, G N Delorenze, R L Hansen, M Kharrazi, J Van de Water, R H Yolken, L A Weiss, L A Croen

**Affiliations:** 1 Division of Research, Kaiser Permanente Northern California, Oakland, CA, USA; 2 Department of Psychiatry and Institute of Human Genetics, University of California, San Francisco, San Francisco, CA, USA; 3 Department of Medical Microbiology and Immunology, University of California, Davis, Davis, CA, USA; 4 MIND Institute, University of California, Davis, Sacramento, CA, USA; 5 Department of Pediatrics, University of California, Medical Health System, Sacramento, CA, USA; 6 Environmental Health Investigations Branch, California Department of Public Health, Richmond, CA, USA; 7 Division of Rheumatology, Allergy and Clinical Immunology, University of California, Davis, Davis, CA, USA; 8 Stanley Division of Developmental Neurovirology, The Johns Hopkins University School of Medicine, Baltimore, MD, USA

## Abstract

Maternal pregnancy levels of the inflammatory marker C-reactive protein (CRP) has been previously associated with autism spectrum disorder (ASD) in the offspring. We conducted a population-based nested case–control study with 500 children with ASD, 235 with developmental delay (DD) and 580 general population (GP) controls to further investigate whether elevated CRP during pregnancy increases the risk of ASD. Maternal CRP concentration was measured in archived serum collected during 15–19 weeks of pregnancy and genome-wide single-nucleotide polymorphism (SNP) data were generated. The levels of CRP were compared between ASD vs GP and DD vs GP. The genetic associations with CRP were assessed via linear regression. Maternal CRP levels in mid-pregnancy were lower in mothers of ASD compared with controls. The maternal CRP levels in the upper third and fourth quartiles were associated with a 45 and 44% decreased risk of ASD, respectively. Two SNPs at the CRP locus showed strong association with CRP levels but they were not associated with ASD. No difference was found between maternal CRP levels of DD and controls. The reasons for the lower levels of CRP in mothers of ASD are not known with certainty but may be related to alterations in the immune response to infectious agents. The biological mechanisms underlying this association remain to be clarified.

## Introduction

Autism spectrum disorder (ASD) is a neurodevelopmental disorder with unknown cause(s) for the vast majority of cases. Previous studies suggest that both genetic^1, 2, 3, 4^ and environmental^5, 6^ factors contribute to the etiology of autism. However, the amount that each factor contributes is the subject of an ongoing debate.^7, 8, 9^ Among the suspected environmental factors of autism, maternal infections during pregnancy have been reported by a number of epidemiological studies.^10, 11, 12, 13, 14, 15^ The studies using animal models have also shown an association between maternal infection during pregnancy and abnormal behaviors in offspring^16, 17^ and suggested that maternal immune activation might be one pathway by which maternal infection can lead to elevated risk of autism in the offspring.^18^ Fever, an acute inflammatory response to various environmental factors including infections, has been suggested to increase the risk of autism and developmental delays.^10, 14^

Acute inflammation is a multisystem process that occurs following injury and/or infection. During this process, immune cells release cytokines, including interleukin (IL)-1, IL-6, tumor necrosis factor-alpha, interferon gamma and others. Such pro-inflammatory cytokines in turn induce the liver to synthesize various acute-phase reactant proteins including C-reactive protein (CRP). The levels of CRP increase very rapidly in response to trauma, inflammation and infection and decrease just as rapidly with the resolution of the condition.^19^ Thus, the measurement of CRP is widely used to monitor inflammatory states, including those that have been suggested to increase the risk of autism.^20^

In addition to variation with inflammatory states, the variation in human levels of CRP has been estimated to be 35–40% heritable in several twin studies.^21^ To identify the genetic determinants of CRP levels, genome-wide association study (GWAS) approaches have been used: the strongest influence on CRP levels is the locus containing the *CRP* gene itself. However, additional loci implicate metabolic pathways as well as innate and adaptive immune responses, including associations near the *IL6*, *IL6R, NLRP3, IL1F10* and *IRF1* genes.^22, 23, 24^

Because association does not always indicate causation, previous studies have utilized a Mendelian randomization approach to leverage genetic information to assess the possible causal relationship of CRP levels with health outcomes. This approach takes advantage of the random allocation of alleles at conception to test the hypothesis that a trait is causally linked to disease. To support causality, the genetic variants that influence the trait should also influence disease risk. For example, this approach has shown that CRP concentration is associated but not causal for coronary heart disease.^25, 26^

Increased levels of CRP during pregnancy have been associated with a number of adverse pregnancy outcomes including preterm birth, low birth weight, small for gestational age and pre-eclampsia.^27, 28, 29, 30, 31^ However, the association between CRP levels during pregnancy and autism has not been intensively studied. The only published study, to our knowledge, reported that elevated levels of CRP (>5.84 mg dl^−1^) during the first and early second trimesters of pregnancy increased risk of ASD by 43%.^32^ However, the study did not investigate whether maternal CRP levels were also associated with other developmental disorders.

The objective of this study was to further examine whether maternal mid-pregnancy CRP levels are associated with increased risk of autism and other developmental delays and whether this association might be influenced by genetic determinants of CRP levels.

## Materials and methods

### Study population

The samples for the present study were derived from the early markers for autism study, a population-based nested case–control study designed to evaluate the biologic markers of susceptibility and exposure in archived maternal mid-pregnancy and neonatal blood specimens from the same mother–baby pairs. The early markers for autism study population was drawn from the cohort of children born in California from 2000 to 2003 to women who were pregnant in Orange, San Diego and Imperial Counties, CA, USA, and who participated in the State's prenatal expanded alpha-fetoprotein screening program. Among women delivering live births in the study counties and birth years, 70% had prenatal expanded alpha-fetoprotein screening program screening. The characteristics of these screened women were similar to the characteristics of all the women in the study counties and birth years with respect to maternal age, education level, race/ethnicity, place of birth, parity and health insurance status (Supplementary Appendix Table 1). Three groups of children were identified: children with ASD, children with developmental delay (DD) but not ASD, and general population controls (GP). Children with ASD or DD were ascertained from the California Department of Developmental Services, which operates a system of 21 regional centers that coordinate services for persons with ASD, mental disability and other developmental disabilities. The GP controls were randomly sampled from the birth certificate files after excluding all past or current department of developmental service/regional center clients and were frequency matched to ASD cases by sex, birth month and birth year.

The early markers for autism study samples were obtained in two phases resulting in two independent sample sets. The first phase included 84 children with ASD, 47 DD and 148 GP children born in 2000 and 2001 in the Orange County. The second phase included 416 ASD, 188 DD and 432 GP children born between 2000 and 2003 in the Orange, San Diego and Imperial Counties, CA, USA. The children in the first phase are not included in the second phase.

### Diagnostic verification

ASD and DD diagnoses were verified by a developmental pediatrician following the Diagnostic and Statistical Manual of Mental Disorders, Fourth Edition criteria by review of information abstracted from medical records following a protocol initially developed by the Metropolitan Atlanta Developmental Disabilities Surveillance Program.^33^ Diagnostic agreements between the developmental pediatrician and the department of developmental services were >90% for ASD and >50% for DD.

### CRP measurements

The maternal mid-pregnancy (15–19 weeks gestation) blood specimens were retrieved from the leftover samples of the prenatal screening specimen archives maintained by the Genetic Disease Screening Program, California Department of Public Health. The CRP measurements for each phase of early markers for autism study samples were conducted at a different laboratory using a different technique. The samples for the first phase were analyzed at Dr Yolken's laboratory at John Hopkins University using a microplate solid phase immunoassay method. The reagents were obtained from IBL America, Minneapolis, MN, USA and the assays were performed as previously described.^34, 35^ Duplicate standards were run on each assay plate and the values below the detection limit were analyzed as 0. The sensitivity of the assay allowed the detection of CRP levels greater than 500 ng ml^−1^. The samples for the second phase were analyzed at Dr Van de Water laboratory at the University of California, Davis via a Milliplex magnetic bead immunoassay (Millipore, Billerica, MA, USA) that utilized the Luminex fluorescent-bead-based technology. The CRP concentrations were calculated with the Bio-Plex Manager software using standard curves derived from the known reference cytokine concentrations supplied by the manufacturer. The sensitivity of this assay allowed the detection of CRP at levels greater than 400ng ml^−1^ and values below the limit of detection were assigned the value of (limit of detection)/2. The Luminex method has been used in numerous previous studies suggesting that the method is both reliable and reproducible.^20, 32, 36^ All the laboratory assays were conducted blinded to case–control status.

### DNA extraction and genotyping

The maternal ASD and GP blood samples from the Phase 2 set were treated for DNA extraction as described in Tsang *et al.*^37^ All the maternal samples were genotyped using the Affymetrix Axiom EUR array by the Genomics Core Facility (GCF) at UCSF, using standard protocols. The Axiom EUR array assays approximately 675 000 single-nucleotide polymorphisms (SNPs) across the genome, and is optimized for genome-wide, gene-based and candidate-SNP coverage.^38^ The genotype calling was carried out using Affymetrix Power-Tools in accordance with the Axiom Genotyping Solution Data Analysis Guide provided by Affymetrix (http://www.affymetrix.com/partners_programs/programs/developer/tools/powertools.affx.). The quality controls were carried out for the genotypes and samples to be included in analysis using PLINK software^39^ as reported in Tsang *et al.*^37^

### Statistical analysis

#### Association between maternal CRP and ASD

We analyzed the two samples separately because they were processed in two different laboratories using two different techniques. We compared sociodemographic factors between ASD, DD and GP groups using chi-square tests for categorical variables and *t*-tests for continuous variables. Among the GP group, we also determined the association between sociodemographic factors and maternal CRP levels. The sociodemographic factors were: child sex, child birth month (calendar month), birth year; and maternal ethnicity, race, place of birth (USA, Mexico, other), age at child birth, weight at blood draw and level of education.

The maternal CRP was first analyzed as a continuous variable after natural log-transformation to normalize the distribution, and then categorized into quartiles and quintiles based on the distribution in the GP group. We conducted crude and adjusted logistic regression analyses to determine the association between maternal CRP levels and ASD or DD. In all categorical analyses, the lowest quartile and quintile were the reference group. Sociodemographic factors associated with both ASD and maternal CRP at *P*=0.05 were used to adjust the crude odds ratio in addition to the matching variables (child sex, birth month and birth year). We also tested whether adjustment for ancestry, as measured by principal components (PCs) derived from GWAS data, altered the results. PC-adjusted models included other potential confounders described above, with the exception of race/ethnicity, which would be accounted for by the PCs. To further explore the association between CRP and ASD, we examined the risk of ASD in the children of mothers with CRP levels ⩾1 mg dl^−1^, a concentration associated with clinical relevance.^19^

#### CRP heritability and genome-wide association analysis

The final set of high-quality SNP markers from ASD and GP control mothers from the phase 2 sample set was used to generate a genetic-relationship matrix and calculate marker-based additive heritability (*h*^2^) of log-transformed CRP levels after exclusion of outliers. A restricted maximum-likelihood model implemented in GCTA software^40^ was utilized with the same sociodemographic covariates as above, plus 10 genetic ancestry principal components and a covariate indicating child phenotype (ASD, DD, GP). The heritability estimate indicates the proportion of the total phenotypic variance in CRP level explained by the polygenic genetic contribution estimated by genotyped markers after the exclusion of the effect of sociodemographic factors and of 10 genetic ancestry principal components.

A GWAS study was performed on CRP levels for the phase 2 ASD and GP control mother set with a linear regression analysis for common SNPs adjusted for the same set of covariates using PLINK software.^39^ The distribution of test statistics has been evaluated with a quantile–quantile plot generated in R 3.2.0 environment (The R Project for Statistical Computing [http://www.r-project.org]) and the top associated genetic loci with a marker that reached genome-wide significance after Bonferroni multiple test correction (cutoff *P*=7.6 × 10^−8^), were considered. Linear and logistic regression models were used to assess the significance of the GWAS top SNPs in a model for CRP levels and for ASD outcome in the R 3.2.0 environment. The linkage disequilibrium among the genetic markers located in the genomic region of associated top SNPs was assessed measuring *r*^2^ using the 1000 Genome Project EUR genetic data as the reference panel.^41^ The regional genomic plots that visualize in a log-scale the association *P*-value and the reciprocal *r*^2^ between the top hit and other genetic markers were drawn using Locuszoom tool.^42^

The study was approved by the institutional review boards of the California Health and Human Services Agency and Kaiser Permanente of Northern California.

## Results

### Sociodemographic factors

The first sample included 84 children with ASD, 46 with DD and 148 GP children. There was no difference between the ASD cases and the GP controls for gestational age and maternal weight at blood draw. However, the mothers of ASD cases were more likely to be non-Hispanic, born in the United States and to be slightly older compared with the mothers of GP controls (Table 1). The second sample included 416 ASD cases, 188 DD and 432 GP. There was no difference between the ASD cases and the GP controls in maternal age, race, gestational age and maternal weight at blood draw. In sample set #1 and #2, maternal ethnicity, race, weight at blood draw and place of birth were associated with level of CRP (Supplementary Appendix Table 2). The mothers of ASD cases were slightly more educated, more likely to be non-Hispanic and born in the United States (Table 1). Only maternal ethnicity was associated with both ASD and CRP at *P*<0.05 but in our final model estimating risk of ASD associated with maternal CRP, we included maternal ethnicity, the matching variables (child sex, birth month and birth year) and maternal weight at blood draw because of its high association with CRP levels.

### Association between maternal CRP and developmental outcome

#### First sample set

Median CRP levels were lower in the mothers of children with ASD (1.28 mg dl^−1^, interquartile range=0.54–3.06) compared with the mothers of GP controls (2.43 mg dl^−1^, interquartile range=0.61–3.82; Supplementary Appendix Figure 1A). We did not find an association between the maternal CRP levels (measured on a continuous scale) and the risk of ASD (crude odds ratio (OR)=0.88, 95% confidence interval (CI)=0.73–1.05; adjusted odds ratio (AOR)=0.97, 95% CI=0.78–1.20). There was no significant difference in the levels of maternal CRP between ASD and GP controls when comparing quartiles or quintiles. We found a similar null result comparing the DD with the GP controls (Table 2).

#### Second sample set

Median CRP levels were significantly lower in the mothers of children with ASD (1.94 mg dl^−1^, interquartile range=1.04–3.90) compared with the mothers of GP controls (2.40 mg dl^−1^, interquartile range=1.32–4.48; Supplementary Appendix Figure 1B). The risk of ASD decreased by 17% for every one unit increase in the level of maternal CRP during pregnancy (OR=0.83, 95% CI=0.73–0.95; AOR=0.83, 95% CI=0.72–0.97; Table 3). The proportion of women with a CRP concentration in the upper third and fourth quartiles was lower among the mothers of children with ASD compared with the mothers of GP controls (third quartile: 19.7% vs 25.3, AOR=0.57, 95% CI=0.38–0.85; fourth quartile: 20.9% vs 27.1%, AOR=0.58, 95% CI=0.38–0.89). We found a similar trend when CRP concentration was categorized into quintiles (Table 3). These results were adjusted for sex, birth month, birth year, maternal ethnicity and weight at blood draw. They remain unchanged if we replace maternal ethnicity with the derived 10 principal components representing genetic ancestry. The CRP values above 1 mg dl^−1^ were not associated with increased risk of ASD (data not shown). There was no significant difference in the levels of maternal CRP between DD and GP controls analyzing CRP as a continuous or categorical variable (Table 3).

**Figure 1:**
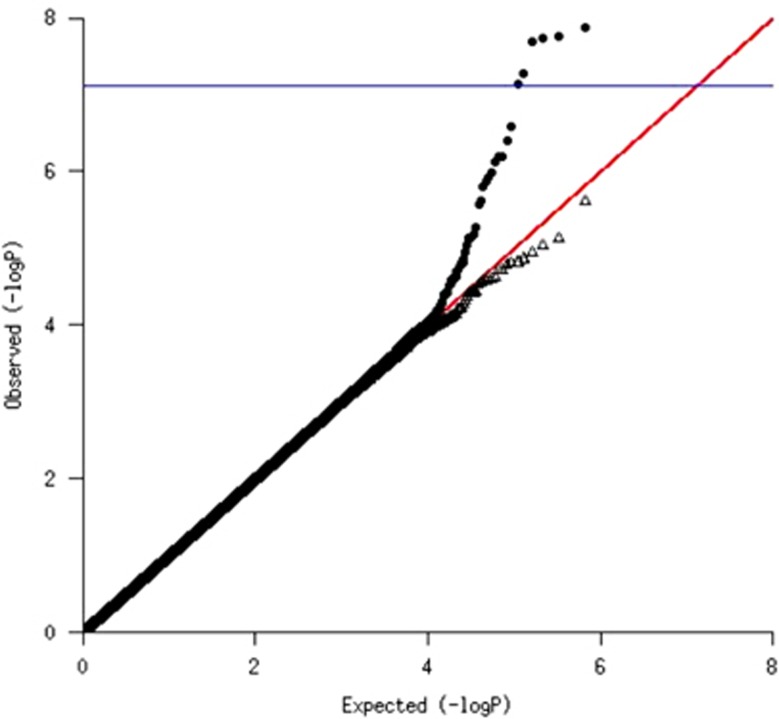
Quantile–quantile plots of the association of genome-wide single-nucleotide polymorphisms (SNPs) with maternal mid-pregnancy C-reactive protein (CRP) levels: SNPs located in CRP locus have been included (filled circles) or excluded (open triangles). The *y* axis shows the negative logarithm of the observed association *P*-value for each tested SNP; the *x* axis shows the negative logarithm of the expected *P*-value based on a statistical sample. The red line is the line of equivalence, observed = expected. The blue line shows the Bonferroni significance threshold for multiple tests (0.05/658 581 SNPs=7.6 × 10^−^^8^).

To determine whether genetic determinants of CRP levels might be driving the association between CRP and ASD, we first assessed the overall genetic signal from the GWAS by examining whether the distribution of test statistics deviated from that expected by chance. In a quantile–quantile plot comparing observed with expected distributions, there was an elevated tail indicating strong genetic signal (Figure 1). The SNP association, which remained significant after Bonferroni multiple testing correction, was within the *CRP* locus itself, along with additional SNPs with suggestive association (*P*<10^−6^). The linkage disequilibrium in the region suggests two semi-independent signals (regional Supplementary Figure S1) (rs3116656; beta=0.30; *P*-value=1.4 × 10^−8^ and rs2794520; beta=−0.25; *P*-value=3.9 × 10^−7^; linkage disequilibrium *r*^2^<0.2). The two SNPs were associated with CRP levels regardless of case–control status (Table 4). After excluding the *CRP* locus, the distribution of test statistics returned to the null expectation (Figure 1) and there was no detectable polygenic SNP heritability for CRP levels (*h*^2^=0; s.e.=0.31). Thus, genetic determination of CRP levels can be adequately summarized by using these two SNP markers.

Incorporating the two SNPs representing the top loci into the regression model attenuated the association between the CRP level and the ASD outcome, but the association remained significant and negative (AOR=0.86, 95% CI=0.77–0.97). When we included an interaction term between CRP and SNPs in the regression models estimating risk of ASD, the *P*-values for the interaction terms were all greater than 0.1 (*P*-value for SNP1 × CRP=0.71 and *P*-value for SNP2 × CRP=0.22). The SNPs that significantly influence the CRP level were not associated with ASD (rs3116656: AOR=0.98, 95% CI=0.92–1.04; rs2794520: AOR=1.02, 95% CI=0.96–1.08).

## Discussion

In the present study, we found no association between maternal mid-pregnancy CRP levels and risk of ASD in the smaller sample, but in the larger sample, the mothers of children with ASD had lower levels of mid-pregnancy CRP compared with those of GP controls. This finding is in contrast to the one prior study that examined CRP levels in association with ASD, which reported higher levels of CRP in the mothers of children with ASD compared with controls.^32^ Our current study and the study by Brown *et al.*^32^ have conflicting conclusions, even though the ranges of CRP levels were similar; the samples were collected at approximately the same period during pregnancy and both the studies used a similar laboratory technique to analyze the serum samples (our sample set 2). In contrast to the Brown study, which was based on an ethnically homogeneous sample from Finland, our sample included a racially and ethnically diverse population. Although the CRP levels have been shown to vary significantly across different ethnic groups,^23^ our finding of a negative association between the maternal CRP level and ASD was observed consistently within each race/ethnic group in our sample. Also in contrast to the Brown study, our study controlled for maternal weight at the time of blood draw, which is also associated with the CRP levels. However, maternal weight did not influence the direction of association that we observed.

Although there is no established consistent baseline levels of CRP, the levels have been shown to increase during normal pregnancy,^29, 43, 44, 45^ and the relationship between CRP level and ASD risk may also vary over the pregnancy. Our results reflect levels of CRP measured between gestational weeks 15–19 weeks only; we were unable to evaluate different pregnancy time periods. One study reported that values above 1 mg dl^−1^ during pregnancy are clinically abnormal.^45^ In our study, we did not find an association between those levels and increased risk of ASD. Hwang *et al.*^29^ reported that CRP levels ranged from 0.04 to 2.03 mg dl^−1^ during the 15–20 weeks of pregnancy, corresponding to the period during pregnancy when maternal samples were collected for our study. Among 99% of control group mothers from the second sample, the CRP values ranged from 0.04 to 22.2 mg dl^−1^. The 10-fold difference in the range of CRP levels between the two studies may be related to the differences in the assays used or other factors related to the study populations.

Further, we investigated whether genetic determinants of CRP levels might explain our association results or establish causality via Mendelian randomization. We found two significant associations with maternal CRP level at the *CRP* gene locus: the best hit rs3116656 is in high linkage disequilibrium (*r*^2^>0.8) with a previously reported CRP-associated SNP in Ridker *et al.*^24^ and the second, rs2794520, replicated a previous finding of Ridker *et al.* These semi-independent SNPs accounted for all the genetic signals that we observed, but did not account for the association between CRP and ASD outcome. Further, the CRP-associated SNPs were not themselves associated with ASD outcome. Thus, an unmeasured factor is likely to be associated with the CRP levels and driving the negative association that we observed between CRP and ASD. However, our Mendelian randomization analysis did not have sufficient power to determine causality for the observed association between CRP and ASD.

In the present study, we did not find an association between maternal mid-pregnancy CRP levels and developmental delay. These findings may suggest that variation in CRP levels during pregnancy may lead to changes that are specific to ASD. However, more studies are needed to confirm the specificity of the association between maternal levels of CRP and ASD.

Infections are the leading cause of acute inflammation. Previously, we found that maternal infections diagnosed in a hospital setting, which may represent cases of more severe infections, were associated with increased risk of ASD compared with infections diagnosed in an outpatient setting.^15^ Atladotir *et al.*^10^ also found that maternal fever lasting more than 7 days was associated with increased risk of ASD as compared with fever that lasted less than 7 days, suggesting that longer periods of inflammation are associated with increased risk of autism. The measured levels of CRP in the present study are more likely to represent baseline CRP at the time of blood collection, as they were normally distributed. The low levels of CRP in the mothers of children with ASD might indicate a possible immune dysregulation. Our present results combined with the previous results of association between infections and autism suggest that some mothers of children with ASD either mount an excessive inflammatory response to environmental factors or they have a baseline immune function that is low and do not mount the appropriate inflammatory response to clear infections or other environmental insults. However, more studies are needed to support these hypotheses.

The results of this study should be considered in light of the following limitations. We were unable to validate the diagnostic status of ASD by systematic clinical evaluation. Instead, we relied on expert review of information recorded as part of diagnostic eligibility for developmental services from regional center records. In addition, we did not have any clinical information such as infection status of the mothers and we were unable to stratify our analysis on maternal obesity status because the variable was not available. Another limitation of the study is that we were not able to stratify our analyses by ASD subtypes. The strengths of the study include the replication of results (although power was not sufficient in the first sample set, the trend was in the same direction as the significant findings in the second sample set) in two separate study populations with samples analyzed in two different laboratories using two distinct methods, and the investigation of genetic determinants of CRP level. All the laboratory assays were conducted blinded to case–control status. Finally, the inclusion of the DD group allowed us to evaluate the specificity of the findings to ASD.

## Conclusion

The maternal CRP levels at 15–19 weeks gestation were lower in the mothers of ASD compared with controls. Although strong genetic determinants of CRP level exist, they do not account for the association between CRP and ASD outcome. The reasons for the lower levels of CRP in the mothers of ASD are not known with certainty but may be related to alterations in the immune response to infectious agents. The mechanisms behind these alterations should be the subject of future investigations.

## Figures and Tables

**Table 1:** Demographic characteristic of ASD cases, DD and GP controls: sample 1 and 2—the early markers for autism study

	*Sample 1*			*Sample 2*		
	*ASD* N=*84 (%)*	*DD* N=*46 (%)*	*GP* N=*146 (%)*	*ASD* N=*416 (%)*	*DD* N=*188 (%)*	*GP* N=*432 (%)*
*Maternal age (years)*						
<20	2.4	6.4	6.1	3.4	13.8	5.3
20–24	8.3	17.0	22.3	13.9	23.0	16.7
25–29	21.4	36.2	31.1	26.2	27.7	30.2
30–34	45.2	27.7	30.4	37.3	23.4	34.7
⩾35	22.6	12.8	10.1	9.2	12.2	13.0
						
*Maternal education*						
<High school	NA	NA	NA	15.1	41.9	24.5
High school	NA	NA	NA	20.0	26.9	26.9
Undergrad college	NA	NA	NA	44.2	25.3	34.0
Post-graduate	NA	NA	NA	19.7	5.9	13.7
						
*Maternal race*						
White	67.9	76.6	79.1	73.6	87.1	78.9
Asian	22.6	17.0	18.2	21.2	6.5	15.5
Other	7.1	6.4	2.7	4.8	6.5	4.9
						
*Maternal ethnicity*						
Hispanics	23.8	57.5	46.6	37.5	69.9	47.0
Non-Hispanics	76.2	42.6	53.4	62.0	30.1	52.1
						
*Maternal place of birth*						
USA	53.6	34.0	44.6	50.5	44.1	48.2
Mexico	10.7	44.7	36.5	11.1	46.2	30.6
Other	35.7	21.3	18.9	13.2	9.7	21.3
						
*Maternal weight at blood draw (lbs)*						
Mean (s.d.)	145.1 (26.7)	149.8 (38.9)	146.5 (34.1)	151.8 (36.0)	158.5 (37.2)	150.8 (34.1)
						
*Gestational age at blood draw (days)*						
Mean (s.d.)	119.8 (7.9)	117.8	118.9 (7.4)	119.1 (8.8)	119.9 (9.2)	118.7 (8.4)
						
*Sex*						
Male	86.9	57.5	87.2	82.5	56.5	82.9
Female	13.1	42.6	12.8	17.6	43.6	17.1
						
*Child birth year*						
2000	27.4	48.9	17.6	17.6	24.2	18.5
2001	72.6	51.1	82.4	25.5	28.5	25.9
2002	NA	NA	NA	41.8	37.1	41.8
2003	NA	NA	NA	15.4	10.2	15.3

Abbreviation: ASD, autism spectrum disorder; DD, developmental delay; GP, general population; NA, not applicable.

**Table 2:** Crude and adjusted odds ratios with their 95% CIs comparing levels of CRP among children with ASD, DD and GP controls—the early markers for autism study: sample 1

	*Developmental outcome*			*ASD* *vs* *GP*		*DD* *vs* *GP*	
	*ASD (*n=*84)*	*DD (*N=*47)*	*GP (*N=*148)*	*Crude OR (95% CI)*	*AOR*^a^ *(95% CI)*	*Crude OR (95% CI)*	*AOR*^b^ *(95% CI)*
CRP as a continuous variable (mean concentration in mg dl^−1^ and s.d.)	2.00 (2.04)	3.09 (2.80)	2.63 (2.33)	0.88 (0.73–1.05)	0.97 (0.78–1.20)	1.09 (0.87–1.37)	1.02 (0.80–1.31)
							
*CRP values by quartile (range in mg dl^−1^)*							
Q1 (0.05–0.615)	24 (28.6)	10 (21.3)	37 (25.0)	1	1	1	1
Q2 (0.616–2.393)	37 (44.1)	14 (29.8)	37 (25.0)	1.54 (0.78–3.06)	1.87 (0.89–3.92)	1.40 (0.55–3.55)	1.26 (0.49–3.25)
Q3 (2.394–3.821)	7 (8.3)	7 (14.9)	37 (25.0)	0.29 (0.11–0.76)	0.36 (0.13–1.00)	0.70 (0.24–2.04)	0.57 (0.19–1.75)
Q4 (3.822–10.253)	16 (19.1)	16 (34.0)	37 (25.0)	0.63 (0.28–1.40)	0.91 (0.35–2.40)	1.50 (0.60–3.77)	1.14 (0.40–3.24)
							
*CRP values by quintile (range in mg dl^−1^)*							
Q1 (0.05–0.455)	18 (21.4)	9 (19.2)	29 (19.6)	1	1	1	1
Q2 (0.456–1.291)	24 (28.6)	8 (17.0)	30 (20.3)	1.29 (0.58–2.86)	1.36 (0.58–3.16)	0.86 (0.29–2.53)	0.79 (0.27–2.36)
Q3 (1.292–2.904)	20 (23.8)	10 (21.3)	30 (20.3)	1.07 (0.48–2.43)	1.37 (0.56–3.32)	1.07 (0.38–3.02)	0.87 (0.29–2.55)
Q4 (2.905–4.390)	6 (7.1)	8 (17.0)	30 (20.3)	0.32 (0.11–0.93)	0.42 (0.14–1.27)	0.86 (0.29–2.53)	0.74 (0.25–2.24)
Q5 (4.391–10.253)	16 (19.1)	12 (25.5)	29 (19.6)	0.83 (0.35–1.96)	1.33 (0.47–3.80)	1.22 (0.44–3.39)	0.83 (0.26–2.68)

Abbreviations: AOR, adjusted odds ratio; ASD, autism spectrum disorder; CI, confidence interval; CRP, C-reactive protein; DD, developmental delays; GP, general population.

a Adjusted for sex, birth month, birth year, maternal ethnicity and weight at blood draw.

b Adjusted for maternal ethnicity, maternal race and weight at blood draw.

**Table 3:** Crude and adjusted odds ratios with their 95% CIs comparing levels of CRP among children with ASD, DD and GP controls–the early markers for autism study: sample 2

	*Developmental outcome*			*ASD* *vs* *GP*		*DD* *vs* *GP*	
	*ASD (*n=*416)*	*DD (*N=*188)*	*GP (*N=*432)*	*Crude OR (95% CI)*	*AOR*^a^ *(95% CI)*	*Crude OR (95% CI)*	*AOR*^b^ *(95% CI)*
CRP as a continuous variable (mean concentration in mg dl^−1^ and s.d.)	3.13 (3.75)	3.93 (3.96)	3.63 (5.16)	0.83 (0.73–0.95)	0.83 (0.72–0.97)	1.14 (0.96–1.36)	0.95 (0.78–1.17)
							
*CRP values by quartile (range in mg dl^−1^)*							
Q1 (0.04–1.32)	147 (35.3)	47 (25.0)	110 (25.5)	1	1	1	1
Q2 (1.33–2.40)	100 (24.0)	42 (22.3)	107 (24.8)	0.70 (0.48–1.01)	0.71 (0.49–1.04)	0.92 (0.56–1.51)	0.75 (0.45–1.26)
Q3 (2.41–4.48)	82 (19.7)	48 (25.3)	106 (24.5)	0.57 (0.39–0.83)	0.57 (0.38–0.85)	1.06 (0.65–1.72)	0.76 (0.45–1.28)
Q4 (4.49–78.68)	87 (20.9)	51 (27.1)	109 (25.2)	0.59 (0.40–0.86)	0.58 (0.38–0.89)	1.10 (0.68–1.76)	0.68 (0.40–1.17)
							
*CRP values by quintile (range in mg dl^−1^)*							
Q1 (0.04–1.00)	103 (24.8)	36 (19.2)	87 (20.1)	1	1	1	1
Q2 (1.01–1.88)	102 (24.5)	30 (16.0)	89 (20.6)	0.98 (0.65–1.47)	0.98 (0.65–1.48)	0.82 (0.46–1.44)	0.69 (0.38–1.24)
Q3 (1.89–3.08)	77 (18.5)	37 (19.7)	85 (19.7)	0.76 (0.50–1.15)	0.83 (0.53–1.29)	1.05 (0.61–1.82)	0.72 (0.40–1.30)
Q4 (3.09–5.04)	64 (15.4)	37 (19.7)	86 (19.9)	0.62 (0.40–0.96)	0.63 (0.39–1.00)	1.04 (0.60–1.80)	0.68 (0.38–1.23)
Q5 (5.05–78.68)	70 (16.8)	48 (25.3)	85 (19.7)	0.69 (0.45–1.06)	0.70 (0.44–1.14)	1.37 (0.81–2.31)	0.83 (0.46–1.50)

Abbreviations: AOR, adjusted odds ratio; ASD, autism spectrum disorder; CI, confidence interval; CRP, C-reactive protein; DD, developmental delays; GP, general population.

a Adjusted for sex, birth month, birth year, maternal ethnicity and weight at blood draw and parity.

b Adjusted for maternal ethnicity, maternal race and weight at blood draw.

**Table 4:** Association between the top two single-nucleotide proteins and maternal mid-pregnancy C-reactive protein–the early markers for autism study: sample 2

*SNP*	*Chromosome*	*Position*	*Major allele*	*Frequency*	*Adjusted odds ratio*^a^ *(95% CI)*
rs3116656	1	159692372	G	0.29	1.35 (1.22–1.49)
rs2794520	1	159678816	T	0.39	0.78 (0.71–0.86)

Abbreviations: CI, confidence interval; SNP, single-nucleotide polymorphism.

a The regression model was adjusted for child case–control status, maternal weight at blood draw, child birth year, birth month, sex and race/ethnicity.
