# Supplementary material for: Maternal mid-pregnancy C-reactive protein and risk of autism spectrum disorders: the early markers for autism study
Source: Transl Psychiatry. 2016 Apr 19;6(4):e783–. doi: 10.1038/tp.2016.46 (PMC4872404; doi:10.1038/tp.2016.46)
Supplement: Supplementary Appendix Figure 1 [file tp201646x1.doc]

| A: Levels of Maternal CRP by case status in sample set #1 | B: Levels of maternal CRP by case status in sample set #2 |
| --- | --- |

Appendix Figure 1: Levels of maternal CRP by case status in both study sample #1 and #2.
