# Supplementary material for: Maternal mid-pregnancy C-reactive protein and risk of autism spectrum disorders: the early markers for autism study
Source: Transl Psychiatry. 2016 Apr 19;6(4):e783–. doi: 10.1038/tp.2016.46 (PMC4872404; doi:10.1038/tp.2016.46)
Supplement: Supplementary Appendix Table 1 [file tp201646x2.doc]

| Appendix Table 1. Demographic characteristics of women who delivered singleton births in San Diego, Orange, and Imperial Counties from January 2000-June 2003: women with prenatal screening are similar to the total population. | | |
| --- | --- | --- |
|  | Prenatal Screening (n=52,523) | All Births  (n=81,105) |
| Maternal age | **%** | **%** |
| <20 | 7.0 | 8.1 |
| 20-24 | 19.3 | 20.2 |
| 25-29 | 28.2 | 26.2 |
| 30-34 | 32.1 | 27.4 |
| 35-39 | 11.6 | 14.8 |
| 40+ | 1.9 | 3.4 |
| Maternal Education |  |  |
| <High School | 25.5 | 27.7 |
| High School | 26.2 | 25.6 |
| College | 35.9 | 34.5 |
| Post Grad | 12.5 | 12.2 |
| Maternal Race/Ethnicity |  |  |
| White–non-Hispanic | 33.9 | 33.5 |
| White–Hispanic: US Born | 15.2 | 14.8 |
| White–Hispanic: non-US Born BBornVBorn | 33.3 | 35.2 |
| Black | 2.7 | 2.9 |
| Asian: US Born | 1.8 | 1.7 |
| Asian: non-US Born | 11.0 | 9.8 |
| Other | 2.3 | 2.2 |
| Maternal Birthplace |  |  |
| California | 36.8 | 39.6 |
| Other US | 13.5 | 13.9 |
| Mexico | 31.2 | 33.2 |
| Other | 18.6 | 17.4 |
| Parity |  |  |
| First-born | 41.7 | 40.2 |
| Second-born | 33.5 | 33.0 |
| Third- or later born | 24.7 | 26.8 |
| Health Insurance Status |  |  |
| Self/ Other | 3.0 | 5.1 |
| Private Insurance | 51.4 | 48.1 |
| Government | 45.6 | 46.8 |
