# Supplementary material for: Maternal mid-pregnancy C-reactive protein and risk of autism spectrum disorders: the early markers for autism study
Source: Transl Psychiatry. 2016 Apr 19;6(4):e783–. doi: 10.1038/tp.2016.46 (PMC4872404; doi:10.1038/tp.2016.46)
Supplement: Supplementary Appendix Table 2 [file tp201646x3.doc]

Appendix Table 2: P values showing Association Between Demographic Factors and Levels of C-reactive protein in Mothers in the Control Group -- the Early Markers for Autism study.

| Demographic Factors | P values for the analysis of variance test | |
| --- | --- | --- |
|  | Sample set #1 | Sample set #2 |
| Maternal weight at blood draw | <0.0001 | <0.0001 |
| Maternal age | 0.42 | 0.727 |
| Gestational age at blood draw | 0.097 | 0.696 |
| Ethnicity (Hispanic vs. non-Hispanic) | 0.0004 | 0.0011 |
| Maternal race | 0.0004 | < 0.0001 |
| Maternal place of birth | 0.0008 | 0.0007 |
